# Direct evidence of electronic ferroelectricity in YbFe_2_O_4_ using neutron diffraction and nonlinear spectroscopy

**DOI:** 10.1038/s41598-021-83655-6

**Published:** 2021-02-19

**Authors:** K. Fujiwara, Y. Fukada, Y. Okuda, R. Seimiya, N. Ikeda, K. Yokoyama, H. Yu, S. Koshihara, Y. Okimoto

**Affiliations:** 1 Graduate School of Natural Science and Technology, Okayama University, 3-1-1 Tsushimanaka, Kita-ku, Okayama, 700-8530 Japan; 2 National Institutes for Quantum and Radiological Science and Technology (QST), 1-1-1 Kouto, Sayo, Hyogo 679-5148 Japan; 3 Department of Chemistry, Tokyo Institute of Technology, 2-12-1 Ookayama, Meguro, Tokyo 152-8551 Japan

**Keywords:** Materials for devices, Materials for optics

## Abstract

We report the first observation of room temperature spontaneous electric polarization in an electronic ferroelectric material, a YbFe_2_O_4_ single crystal. The observation was based on second harmonic generation (SHG), a nonlinear optical process. Tensor analysis of the SHG signal revealed that this material has a polar charge superstructure with Cm symmetry. This result settles the long-term discussion on the uncertainty about electronic ferroelectric properties, including the charge order structure. We present a complete picture of the polar charge ordering of this material via consistent results from two different characterization methods. The SHG signal shows the same temperature dependence as the superlattice signal observed in neutron diffraction experiments. These results prove ferroelectric coupling to electron ordering in YbFe_2_O_4_, which results in electronic ferroelectricity which is enabled by the real space ordering of iron cations with different valences. The existence of electronic ferroelectricity holds promise for future electronics technologies where devices run a thousand times faster than frequency of the present CPU (a few gigahertz) embedded in smartphones, etc.

## Introduction

Ferroelectric materials have been the basis of electronics technologies and are widely used in modern electronic devices^1^. Essentially, the ferroelectric phenomenon is caused by a spontaneous electric polarization that originates from the spontaneous displacement of polar ions. Thus, ferroelectrics function via the collective motion of ions, i.e., optical phonons and their freezing process. With the development of modern electronics technology, the operating frequency of ferroelectrics must be increased and their driving electric field reduced to realize high-speed and low-energy devices. Additionally, conventional phonon-driven ferroelectrics must overcome the fatigue effects that arise from repeated polarization reversals and ferroelectricity degradation^2,3^.

In 2005, a novel type of ferroelectric material was proposed in which the spontaneous polarization mainly originates from the polar ordering of electrons rather than ionic atoms. Such materials are called “electronic ferroelectrics”^4,5^ and are expected to exhibit unique ferroelectric characteristics such as a low coercive field, excellent durability, and an ultrafast response^6^. In general, the kinetic energy required for an electron system is less than that for a phonon system. Therefore, if an electron-driven polarization mechanism for electronic ferroelectrics is confirmed, it might enable new technologies for high-speed and ultralow energy electronics without the inherent fatigue effects of conventional ferroelectrics.

Two-dimensional rare-earth ferrites *R*Fe_2_O_4_ (*R* = Yb, Lu, Y, etc*.*) have been proposed as candidates for such electronic ferroelectrics. These *R*Fe_2_O_4_ materials have the polar ordering of divalent and trivalent iron cations, Fe^2+^ and Fe^3+^, meaning that polarization switching can only be realized by electronic motion^4,7^. LuFe_2_O_4_ has exhibited charge ordering of Fe^2+^ and Fe^3+^ below T_CO_ ~ 330 K and ferromagnetic iron spin order was observed below T_N_ ~ 245 K in the doubly-stacked iron triangular layers called “W-layers”^4,5^. The interrelated electrical and magnetic properties arising from the strongly-correlated iron electrons constitute a research topic in condensed matter physics and materials science. From the perspective of ferroelectric physics, a pyroelectric current driven by a cooling electric field, a large dielectric constant, and spontaneous polarization, were reported for the first time in 2005^4^.

These findings indicated that the ferroelectric behavior of these materials is related to the charge ordering of Fe^2+^ and Fe^3+^ within the W-layers^4,7^. This implied that not only LuFe_2_O_4_ but also other rare-earth ferrites could be considered an electronic ferroelectric, wherein the electrons in the iron cations and the valence superstructure play an important role in the origin of spontaneous electric polarization. Thus, electronic ferroelectrics are expected to exhibit novel ferroelectric functions different from conventional ferroelectrics.

Some interesting characteristics of these materials have been reported. For example, their small bandgap of approximately 300 meV was found to be caused by electron hopping in the W-layers^7,8^. Ultrafast disruption of the charge ordering by light was also reported^11^. These results showed that these materials have potential application in novel ferroelectric or optical devices, where polarization switching in a low coercive field and ultrafast responses via electron hopping can be utilized. Thus, *R*Fe_2_O_4_ materials have attracted much attention as prototypes of electronic ferroelectrics arising from charge ordering at room temperature^4,9^.

These observations of the favorable electronic ferroelectric properties of *R*Fe_2_O_4_ triggered a resurgence of studies on these rare-earth ferrites. Therefore, the crystal structure and ferroelectricity of LuFe_2_O_4_ were critically re-examined. However, the high electrical conductivity due to electron hopping in *R*Fe_2_O_4_^8,9^ made the usual experimental methods for studying ferroelectrics difficult (see Supplementary Fig. S1). Ferroelectric properties are typically measured based on the electric field dependence of the polarization, the so-called *P–E* loop observation. In the case of *R*Fe_2_O_4_, the high electrical conductivity prevents these typical *P–E* loop measurements^10,11^. Even if a *P–E* loop can be observed, the possibility of the banana curve^12^ or dielectric breakdown^13^ cannot be dismissed. Furthermore, de Groot et al.^14,15^ conducted X-ray diffraction measurements and, based on bond valence sum analysis, suggested that the iron ordering had inversion symmetry between the W-layers. Moreover, they suggested that LuFe_2_O_4_ belongs to the C2/m space group and further estimated that LuFe_2_O_4_ is neither polar nor ferroelectric.

**Figure 1:**
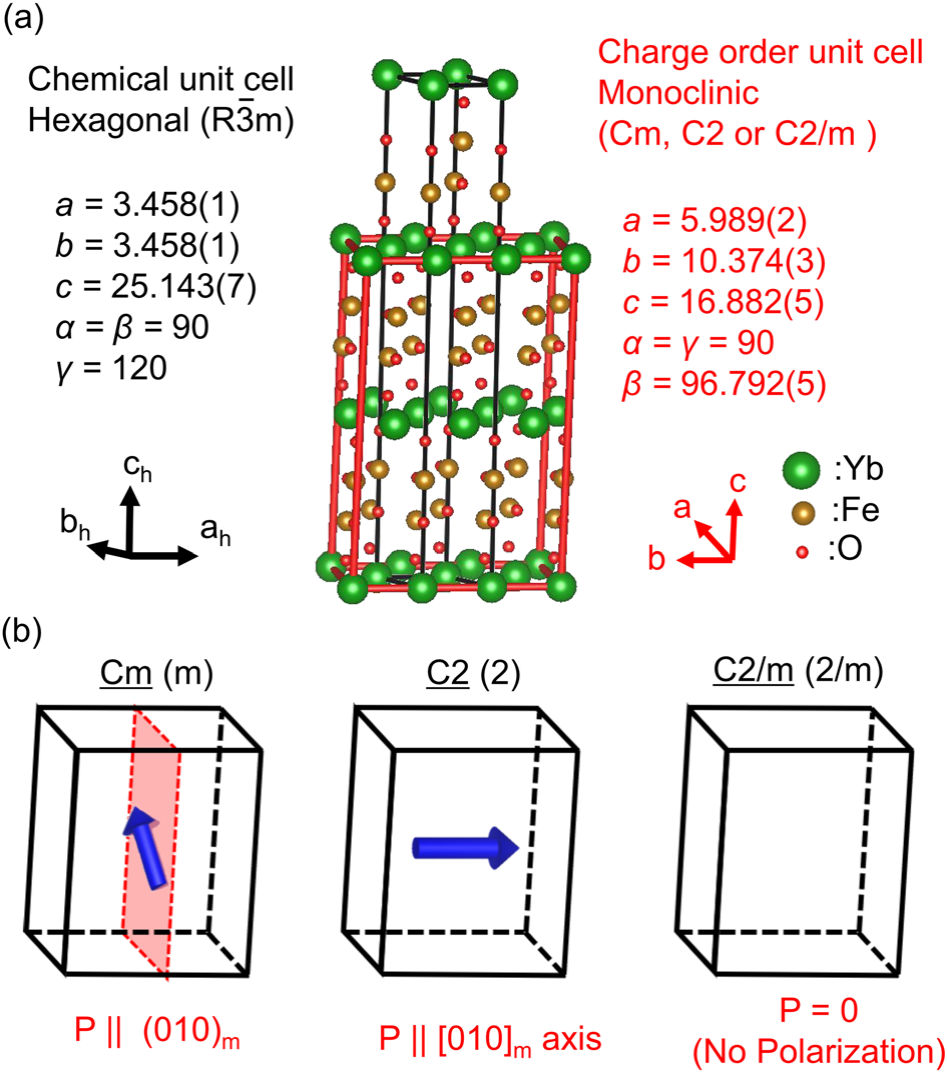
Crystal structure and symmetry of YbFe_2_O_4_. (**a**) Crystal structure of YbFe_2_O_4_. The black lines represent the chemical unit cell with a hexagonal structure (R3m) and the red lines denote the charge order unit cell with a monoclinic structure, which could have Cm, C2, or C2/m symmetry. The unit axes for the hexagonal cell (black) and the monoclinic cell (red) are included as insets. (**b**) The three candidates for the YbFe_2_O_4_ space group: Cm, C2, and C2/m. The blue arrow represents the polarization directions for Cm and C2.

Another experimental challenge to consider when working with these materials is the difficulty of achieving the correct oxide chemistry in rare-earth ferrites^16–19^. It has recently been shown that the charge order structure and, therefore, the electrical and magnetic properties in these materials are highly sensitive to the stoichiometry of not only the oxygen but also the iron content^20^. For example, the superlattice reflection rule of 1/3 1/3 *L*_h_ in hexagonal coordinates, which characterizes the order parameter of the iron charge ordering, is highly dependent on the quality of the crystal or the chemical stoichiometry of *R*Fe_2_O_4_. In particular, Fujiwara et al*.* demonstrated that the Fe sites in *R*Fe_2_O_4_ crystals are prone to cause atomic deficiency, and compensation for iron evaporation during crystal growth is essential to obtain the stoichiometric crystal of YbFe_2_O_4_^20^. A subsequent X-ray diffraction measurement of this stoichiometric YbFe_2_O_4_ crystal showed that its Laue group has "2/m" symmetry based on the extinction rule of superlattice spots^21^, which is consistent with de Groot’s results mentioned above^14,15^. Figure 1a displays the YbFe_2_O_4_ monoclinic unit cell derived from analysis of the observed diffraction extinction rule of the charge ordering. However, even under these considerations, three possible space groups exist, i.e., Cm (polar), C2 (polar), and C2/m (nonpolar), as depicted in Fig. 1b. Therefore, some uncertainty regarding the mechanism of electronic polarity in YbFe_2_O_4_ still exists.

The purpose of this report is to present the first evidence of the polar charge order structure of YbFe_2_O_4_ for the stoichiometric YbFe_2_O_4_ single crystal based on second harmonic generation (SHG) spectroscopy. SHG is a second-order nonlinear effect that can be used to determine whether a crystal possesses inversion symmetry. In addition, this method of optically investigating the charge-ordered state is an ideal non-electric current approach that can sense the states of electrons with small perturbation^22^. We further discuss the relationship between the polar structure and iron charge ordering, which is essential for understanding electronic ferroelectric nature of YbFe_2_O_4_. We also consider the temperature dependence of SHG and neutron diffraction.

## Results

**Figure 2:**
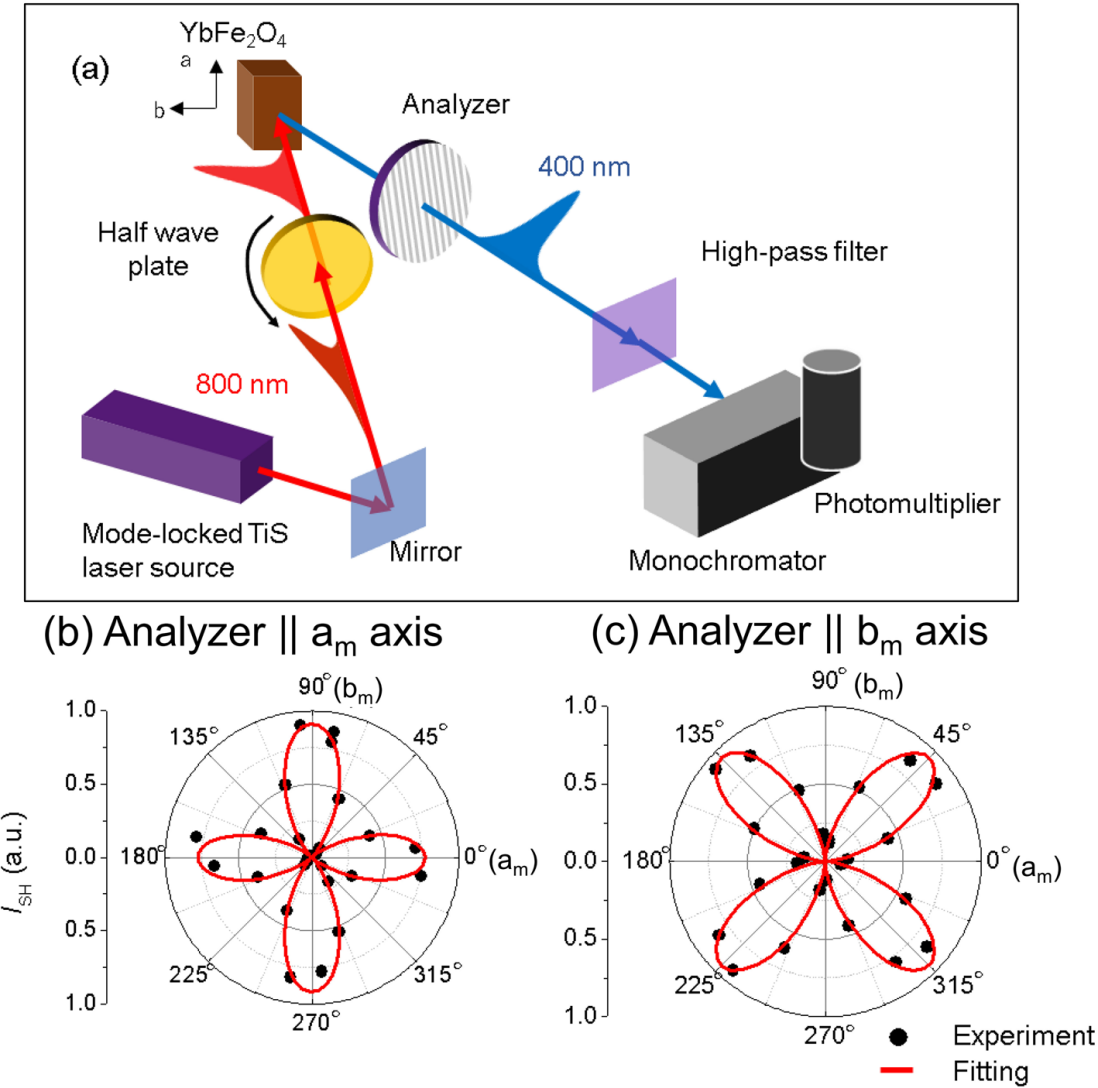
(**a**) Schematic diagram of the optical setup for the SHG polarization angle dependence measurement in the 00*L*_h_ (axis inset) of YbFe_2_O_4_. The red line represents incident light and the blue line represents reflected light. (**b**,**c**) Angle dependence (*θ*) of the SHG measurement. The black circles represent the experimental measurements and the red lines shows the fitting results based on the space group Cm.

Figure 2b,c show the SHG dependence on the optical polarization angle (*θ*) of the incident laser pulse. The angle *θ* is defined as the angle between the incident optical polarization direction and the *b*_m_-axis. 0° and 90° correspond to the *b*_m_- and *a*_m_-axes, respectively, in the monoclinic coordinates of the charge superstructure, as shown in Fig. 2a. The analyzer is positioned after the crystal and oriented along the *a*_m_-axis in Fig. 2b and along the b-axis in Fig. 2c. In both configurations, the *θ* dependence shows four clear leaves, with the directions of each leaf coinciding with the *b*_m_- and *a*_m_-axes of the charge superstructure in monoclinic coordinates. These results are the first evidence of the polar structure of the YbFe_2_O_4_ crystal. As such, the C2/m symmetry proposed in a previous report^23^ can be excluded.

One may question whether the SHG signal obtained in the reflection configuration in Fig. 2a originates from the crystal surface instead of the W-layers. A finite SHG signal at 90° is observed, as shown in Fig. 2b (the surface SHG is forbidden in the S_in_–S_out_ configuration), whereas the minimum signal is at 0° in Fig. 2c (the surface SHG is allowed in the P_in_–P_out_ configuration). These results indicate that the surface SHG is negligible compared to the observed signal for this measurement, meaning that the results obtained represent the polarization behavior W-layers of the material.

We analyzed this *θ* dependence based on the second-order nonlinear susceptibility (χ^(2)^) deduced from the crystal symmetry^24^. In general, the second-order nonlinear polarization (***P***^*NL*^) is described as ***P***^*NL*^ = χ^(2)^:***EE***, where ***E*** is the electric field of the incident light, and the SHG intensity is proportional to |***P***^*NL*^|^2^. As previously indicated, X-ray diffraction analysis suggests that the possible space groups without inversion symmetry are Cm or C2 in the monoclinic structure^21^. However, in space group C2, the symmetry requires the polarization to be along the *b*_m_-axis, which does not account for the observed *θ* dependence of the SHG in Fig. 2. Thus, we analyze Fig. 2b,c based on the only remaining possible space group, Cm. For this symmetry, the contracted tensor of χ^(2)^ (*d*_*i,j*_) is described as follows:

1 $$ d_{i,j} = \left( {\begin{array}{*{20}c} {d_{11} } & {d_{12} } & {d_{13} } & 0 & {d_{15} } & 0 \\ 0 & 0 & 0 & {d_{24} } & 0 & {d_{26} } \\ {d_{31} } & {d_{31} } & {d_{33} } & 0 & {d_{35} } & 0 \\ \end{array} } \right) $$

The *a*_m_- and *b*_m_-axis coordinates for the representation of χ^(2)^ are displayed in the inset of Fig. 2a. Under these conditions, the generated ***P***^NL^ of the crystal is *P*_*a*_^NL^(*θ*) $\propto$ *d*_11_*E*^2^cos^2^*θ* + *d*_12_*E*^2^sin^2^*θ*. Further, *P*_*b*_^NL^ $\left( \theta \right){ } \propto$ 2*d*_26_*E*^2^sin*θ* cos*θ* where *P*_*a*_^NL^ and *P*_*b*_^NL^ are the *a*_m_- and *b*_m_-axis components of ***P***^NL^, respectively, extracted using the analyzer (see Fig. 2a). The red lobes in Fig. 2b,c show the analysis results based on fitting with the Cm symmetry. These results strongly indicate that the YbFe_2_O_4_ space group belongs to Cm with polarization in the (010)_m_ plane. According to the fitting analyses, we can estimate the ratio among some of the χ^(2)^ components (i.e., *d*_11_:*d*_12_:*d*_26_ = 1:−1.1:1.2).

**Figure 3:**
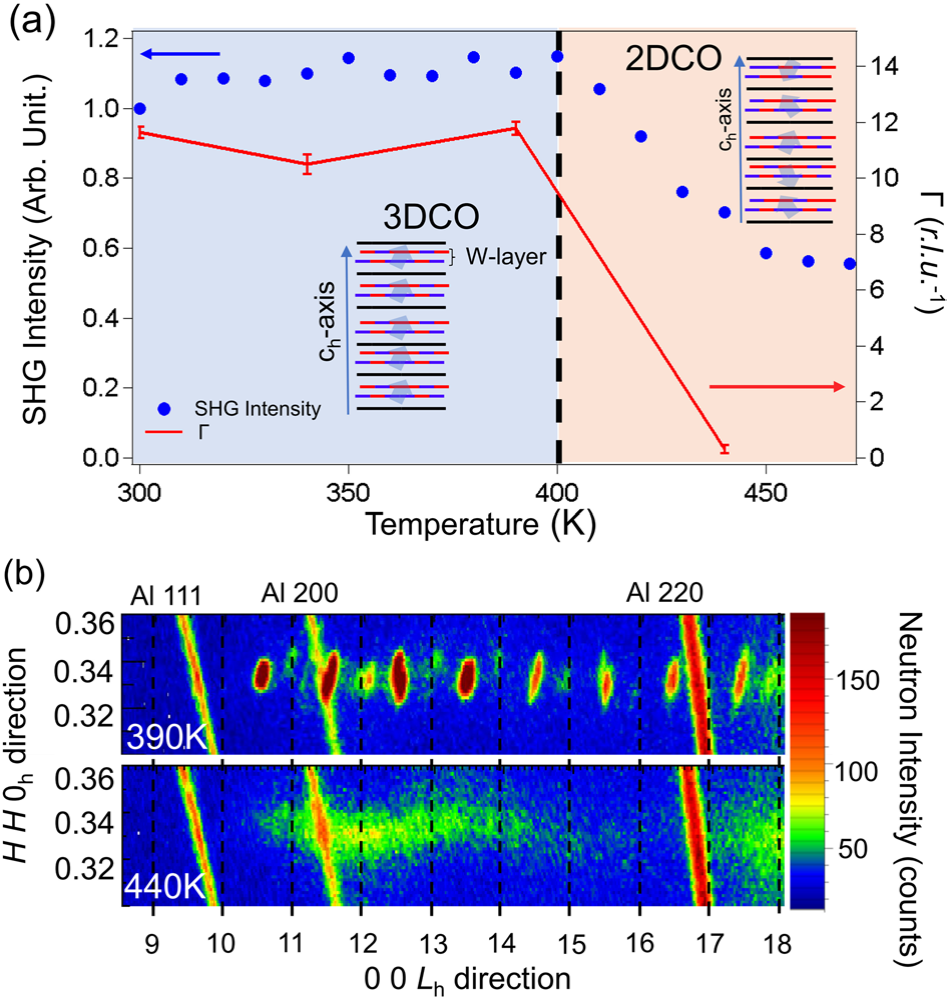
(**a**) Temperature dependence of the SHG signal (*I*_SH_, blue) and correlation length of the charge order (*Γ*, red), estimated from the 1/3 1/3 13.5_h_ peak width along the 0 0 *L*_h_ direction. The dotted black line at 400 K represents 3-dimensional charge order transition temperature (T_CO3D_). The insets labeled 3DCO and 2DCO represent 3-dimensional charge order and 2-dimensional charge order, respectively. (**b**) Reciprocal lattice space mapping of the superlattice diffraction near 1/3 1/3 *L*_h_ in stoichiometric YbFe_2_O_4_ at 390 K (top) and 440 K (bottom). The streaks labeled Al 111, Al 200, and Al 220 are due to diffraction from the sample holder. The colors encode the neutron intensity, with blue for low intensity and red for high intensity.

The final experimental question to discuss is the relationship between the breaking of the inversion symmetry and the iron ordering in the W-layers. Figure 3b presents a contour plot of the neutron diffraction at 390 K and 440 K in the stoichiometric YbFe_2_O_4_ crystal near 1/3 1/3 *L* + 1/2_h_, where *L* is an integer in hexagonal coordinates. Some streaks, labeled Al 111, Al 200, and Al 220, are due to diffraction from the aluminum sample case. At 390 K, distinct superlattice structures are observed at 1/3 1/3 *L* + 1/2_h_, indicating the real-space Fe^2+^ and Fe^3+^ ordering in the W-layers^25^. At 440 K, in contrast, the profiles become rod-like in the [0 0 1]_h_ direction. This indicates dimensional crossover of the iron ordering, i.e., suppression of the charge order correlation between the W-layers. The long-range ordering of the Fe cations within these layers is maintained at this elevated temperature, though.

To investigate the temperature dependence of the *c*-axis correlations in more detail, we evaluated the peak widths of the superlattice reflections. Figure 3a shows the temperature dependence of the inverse observed peak widths (red lines) for the 1/3 1/3 13.5_h_ signal profile, together with the SHG intensities (blue circles). *Γ*, the correlation length along the *c*-axis, shows a sudden decrease above 400 K due to the aforementioned suppression of the charge order coherence length between the W-layers. The blue circles show the temperature dependence of the SHG signal generated from the (00*L*)_h_ plane. The SHG intensity exhibits a minor change between 300 and 400 K and then abruptly decreases above 400 K, similar to the temperature dependence of *Γ*. This result shows that the SHG signal is correlated with the coherence length of the charge ordering along the [001]_h_ of the iron layers, rather than with the crystal symmetry itself.

To understand this behavior, we consider the origin of the temperature dependence of the SHG signal according to the dimensional crossover of the charge ordering. For Cm symmetry, polarization exists within the (010)_m_ plane. As such, the decrease in *Γ* above 400 K indicates suppression of the coherence length in the polar region that generates an SHG signal from each W-layer. This suppression causes partial cancelation of the polarization due to the random orientations of the polarization along the [001]_h_, as shown in the schematics of Fig. 3a, even though the ordering pattern hardly changes^23^. Thus, the suppression of the *c*-axis correlation reduces the SHG intensity. Notably, as the temperature increases to 450 K, the SHG signal persists, whereas the [001]_h_ correlation is largely suppressed. In YbFe_2_O_4_, the penetration depth of 3.1 eV light is approximately 50 nm based on an optical measurement^26^. The residual SHG signal implies not only that the cancelation of the SHG signal is imperfect within the penetration depth but also that the correlation of the charge ordering in the (001)_h_ plane is still robust at higher temperatures of approximately 500 K.

## Discussion

**Figure 4:**
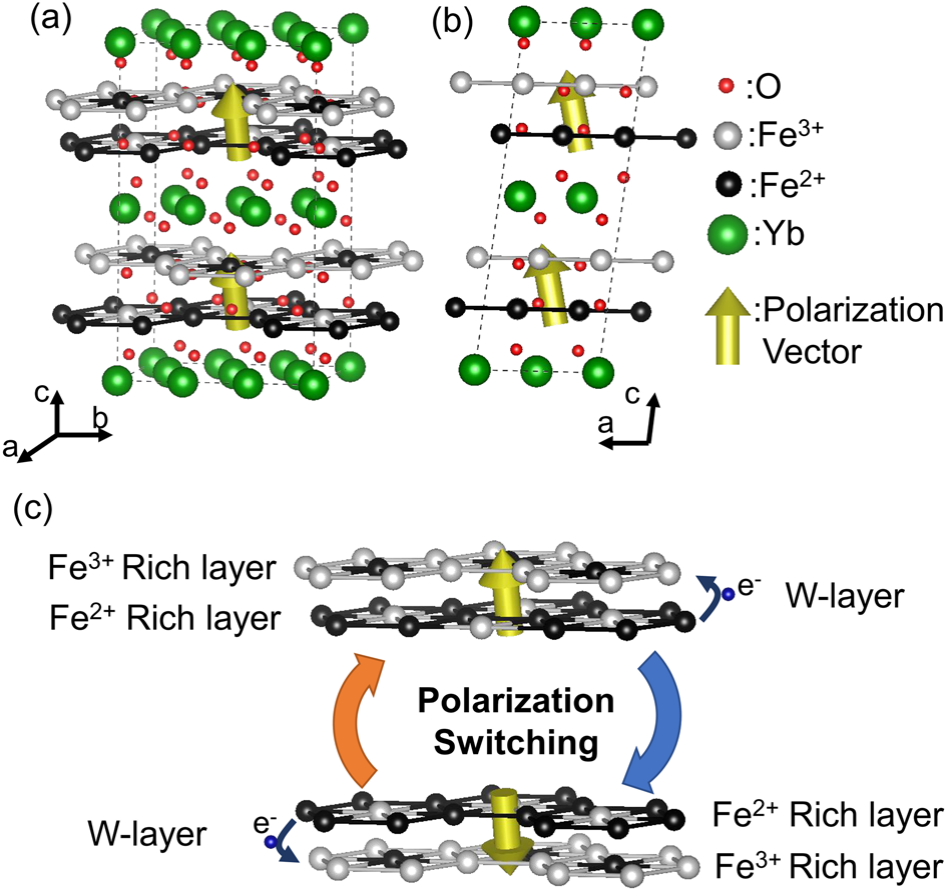
(**a**) Schematic representation of the crystal structure for the Fe^2+^ (black circles) and Fe^3+^ (gray circles) ordering in YbFe_2_O_4_, as expected from the SHG and neutron diffraction measurements. The dotted lines represent the monoclinic unit cell and the red and green colored circles represent the locations of O and Yb, respectively. The yellow arrows denote the polarization direction, which is tilted 16.5° from the [001]_h_. (**b**) Projection of the crystal structure onto the (010)_m_ plane, where the polarization tilt direction can be more clearly resolved. (**c**) Schematic view of the polarization switching due to electron hopping within the W-layers. The possible directions of electron (e^−^, small blue circles) hopping are labeled with small blue arrows. The large orange and blue arrows represent polarization switching. There crystal structure was drawn by VESTA 3 software^27^.

Now that we understand the relationships between charge order structure and SHG intensity in this material, we present a complete picture of the electronic ferroelectric YbFe_2_O_4_ crystal structure and ordering patterns for the first time. In Fig. 4a, we summarize the crystal structure and the Fe^2+^ and Fe^3+^ ordering pattern in YbFe_2_O_4_ based on the monoclinic Cm symmetry, which was proven by the SHG signal and the 1/3 1/3 *L*_h_ superlattice peaks observed in the neutron diffraction pattern. In the depicted structure, Fe^2+^ (black circles) and Fe^3+^ (gray circles) coexist uniformly within the W-layers, resulting in a polar structure. Assuming fixed point charges as in the atomic positions of Fig. 1, we can estimate the magnitude of the spontaneous electric polarization to be 12.9 μC/cm^2^ in YbFe_2_O_4_.

As presented in Fig. 1b, for Cm symmetry polarization exists within the (010)_m_ plane with its direction tilted away from the [001]_h_. According to the point charge model, the tilted angle deviation from the c-axis can be estimated as 16.5°, shown using yellow arrows in Fig. 4a,b. The direction of this polarization is potentially switchable, but only by electron hopping within the W-layers. Applying an external electric field along the [001]_h_ causes polarization due to the charge imbalance between Fe^2+^ and Fe^3+^. As a result, the polarization direction switches in the (010)_m_, as shown by the yellow arrows in Fig. 4c. In contrast to ordinary ferroelectrics, this can be achieved without a structural change. This could facilitate the implementation of novel ferroelectric functions, for example, polarization or polarized domain reversal with less fatigue.

## Conclusion

In summary, we report the first evidence of the polar structure of a stoichiometric YbFe_2_O_4_ crystal based on SHG spectroscopy and neutron diffraction. From the polarization analysis of the SHG signal, the point group (Cm) and the charge order structure were uniquely determined.

Furthermore, a decrease in the SHG intensity was observed above approximately 400 K. This temperature corresponds to the dimensional crossover of the charge order estimated via the neutron diffraction measurements, indicating that this SHG signal originated from the real-space Fe^2+^ and Fe^3+^ ordering. Thus, *R*Fe_2_O_4_ materials can be considered electronic ferroelectrics driven by the electron order, without any structural phase transition. These results could possibly enable novel ferroelectric properties such as a low coercive field, excellent durability with low fatigue, and an ultrafast optical response.

## Method

A single crystal was synthesized using the floating melting method. Fe_2_O_3_ (5N, Rare Metallic Co., Ltd.) and Yb_2_O_3_ (4N, Nippon Yttrium Co., Ltd.) were used as raw materials. To synthesize the sample, 10% excess Fe_2_O_3_ was added to the Yb_2_O_3_ to obtain a stoichiometric crystal. The growth rate and melting temperature were 5 mm/h and 1600 °C, respectively, in a CO/CO_2_ atmosphere. The stoichiometry of the synthesized sample was evaluated via X-ray fluorescence analysis (XRF) and thermo gravity analysis (TGA). The Fe/Yb ratio of the sample was estimated from the integral intensity of the Fe-Kα and Yb-Lα fluorescence line. The ratio of fluorescence intensity had been calibrated with standard samples that were a mixture of Fe_2_O_3_ and Yb_2_O_3_ powders. The error of the calibration line was estimated at less than 0.2%. TGA was obtained from DSC7300 (Hitachi High-Tech Co., Ltd.) under a dynamic flow of dry air (200 ml/min) and the sample was annealed 650 °C (8 h). The sample and reference (α-Al_2_O_3_) was pre-annealed in 120 °C (2 h) in Ar gas atmosphere to remove moisture. The result of XRF and TGA are shown in Table 1. This result was consistent with those reported in Ref.^20^. No impure phases were confirmed in the powder X-ray diffraction pattern using Ulutima IV (RIGAKU Co., Ltd.). The extinction rule for the X-ray diffraction observation of the crystal was observed with four-axis diffractometor HUBAR-512 with Mo–Kα and control software SPEC was used to measure the Bragg spots mapping. (003)_h_, (009)_h_, (110)_h_ and (101)_h_ spots were used to calculate UB matrix. These X-ray diffractions were measured at room temperature (RT). This protocol is almost the same as that in Ref.^24^.

**Table.1:** The sample stoichiometry of YbFe_2_O_4_.

Raw material rate Fe_2_O_3_/Yb_2_O_3_ (mol)	Fe/Yb rate (evaluated by XRF)	Stoichiometry of oxygen YbFe_x_O_y_ (evaluated by TGA)	Average of Fe valence
2.00	1.79(3)	x = 1.79, y = 3.775(5)	2.542(5)
2.20	1.95(3)	x = 1.95, y = 3.960(5)	2.523(5)

Using a diamond cutter ((010)_m_ and (100)_m_) and cleavage ((001)_h_), the fabricated crystal was cut into dimensions of 3 × 3 × 1 mm^3^, in which the largest plane was the (001)_h_. Subsequent SHG measurements were performed on this *ac* plane. Figure 2a shows a schematic representation of the experimental setup used for the SHG measurements. A regenerative amplified mode-locked Ti:sapphire laser system QUANTRONIX INTEGRA (pulse width of 150 fs, repetition rate of 1 kHz, and photon energy of 1.55 eV) was used as the light source. The crystal was irradiated with the laser pulse and the SHG pulse signal produced by the crystal was detected using a high pass filter. During the measurement process, the incident pulse polarization was manipulated using a half-wave plate. The SHG signal was measured in the polarization direction along [010]_m_ (Fig. 2b) and [100]_m_ (Fig. 2c) using the analyzer. To investigate the temperature dependence of the SHG signal, the crystal was set in a vacuum within a cryostat (Oxford MicrostatHe).

Neutron diffraction was performed at the WOMBAT beamline, ANSTO. The reciprocal space of *h h l*_h_ was observed with a 2-dimensional neutron detector. The sample crystal was 3 mm in diameter and 15 mm in length. The incident wavelength was 2.41 Å, and the beam size was 15 mm (horizontally) and 30 mm (vertically). The magnetism was confirmed to be almost the same as that of the SHG sample. Based on the scattering angle *2θ*, the sample rotation angle with respect to the incident beam, ω, and the intensity were recorded and processed. The diffracted intensity was summed along the *H −H* 0_ h_ direction in reciprocal space (corresponding to the height of the 2D detector) to amplify the signal intensity. The reciprocal map of the *HH*0_h_*–*00*L*_h_ slice was processed from these data using LAMP software^28^. The details of neutron diffraction of stoichiometric YbFe_2_O_4_ are described in Ref.^23^.

## Supplementary Information

Supplementary Information.
