# Supplementary material for: Direct evidence of electronic ferroelectricity in YbFe2O4 using neutron diffraction and nonlinear spectroscopy
Source: Sci Rep. 2021 Feb 19;11:4277. doi: 10.1038/s41598-021-83655-6 (PMC7896071; doi:10.1038/s41598-021-83655-6)
Supplement: Supplementary file 1 — Supplementary Information. [file 41598_2021_83655_MOESM1_ESM.docx]

**SUPPLEMENTAL INFORMATION**

**Direct Evidence of Electronic Ferroelectricity in YbFe_2_O_4_ Using Neutron Diffraction and Nonlinear Spectroscopy**

K. Fujiwara, Y. Fukada, Y. Okuda, R. Seimiya, N. Ikeda, K. Yokoyama, H. Yu, S. Koshihara, and Y. Okimoto^*^

**APPENDIX. Temperature dependence of the resistivity**

The electric resistance measurements were done in a vacuum chamber to prevent the sample oxidization. The sample was put on a glass epoxy plate of 1 mm thickness, and they are set on a Cu thermal anchor. The temperature of the thermal anchor is automatically controlled by Cryo-Con 32B. The resistivity was measured by ordinary two wires method by Keighley 2450 source measure unit. The current for the measurement was set at 1μA. The sample size is 2.05 mm [110]_h_ × 2.15 mm [1-10]_h_× 1.15 mm [001]_h_. Au paste was used for electrodes.

The temperature dependence of resistivity was shown in In Fig. S1. As temperature decreases, the resistivity increases, indicating insulating behavior. The in-plane resistivity (E||c_h_) is higher than those in out of plane (E||a_m_ or b_m_). The resistivity shows about 100~1000 Ωcm in 300 K and this value is lower than that of ordinary dielectric materials, relatively. Thus, it is difficult to exactly measure P-E hysteresis measurement. In SHG and neutron diffraction experiments, as mentioned in the text, the charge order shows dimensional crossover at around 400 K. In the low temperature region, the electrical resistivity is sufficiently high to measure the P-E hysteresis curve, however, the large coercive electric field in the low temperature region makes the measurement difficult.


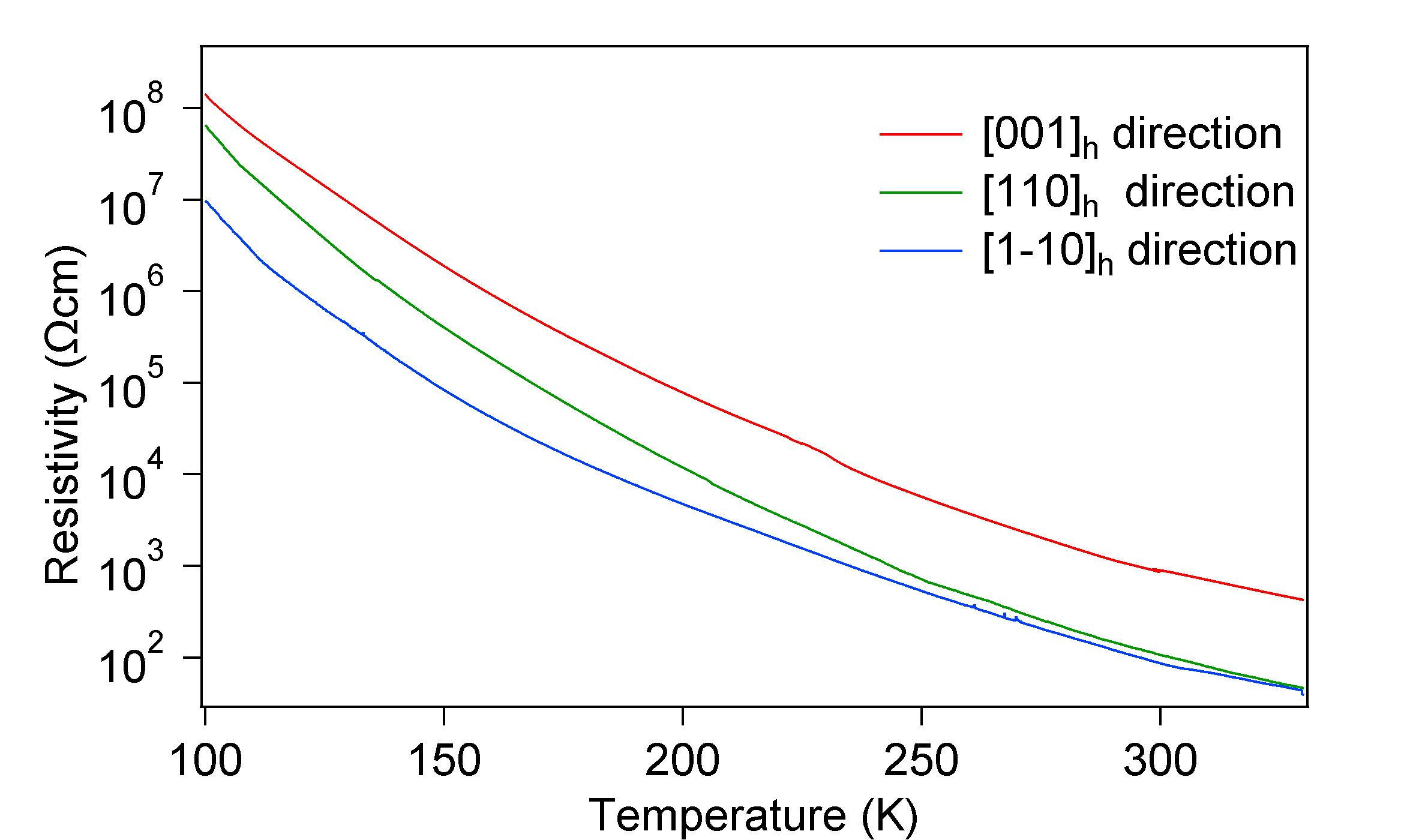


Fig. S1 Temperature dependence of resistivity in stoichiometric YbFe_2_O_4_.
